# School Closures on Bullying Experiences of Treatment-Seeking Children and Youth: The Influence of the COVID-19 Pandemic Within Ontario, Canada

**DOI:** 10.3390/ijerph21121673

**Published:** 2024-12-15

**Authors:** Shannon L. Stewart, Abigail Withers, Jeffrey W. Poss

**Affiliations:** 1 Faculty of Education, Western University, 1137 Western Rd, London, ON N6G 1G7, Canada; sstewa24@uwo.ca; 2 School of Public Health Sciences, University of Waterloo, 200 University Ave W, Waterloo, ON N2L 3G5, Canada; jwposs@uwaterloo.ca

**Keywords:** bullying, victimization, COVID-19, school closures, mental health

## Abstract

Amongst school-aged children and youth, bullying is a significant problem warranting further investigation. The current study sought to investigate the influence of the COVID-19 pandemic waves and school closures on the bullying experiences of 22,012 children aged 4–18-years-old who were referred and assessed at mental health agencies in Ontario, Canada. Individual, familial, and mental health variables related to bullying experiences were also investigated. Data were collected from January 2017 to February 2022. The pre-pandemic period of study included January to June 2017, September 2018/2019 to June 2019/2020. The pandemic period was divided into categories of remote learning (17 March 2020 to 30 June 2020, 8 January 2021 to 16 February 2021, 12 April 2021 to 30 June 2021) and in-person learning (remaining pandemic dates). The summer holidays pre-pandemic were in July–August 2017, 2018, 2019 and during the pandemic they were in July–August 2020 and 2021. Logistic regressions were conducted to analyze data. Findings related to COVID-19 showed bullying rates to be lower during the pandemic when compared to pre-pandemic levels (bullied others during pandemic in school: OR = 0.44, CI = 0.34–0.57; victim of bullying during pandemic in school: OR = 0.41, CI = 0.33–0.5). Furthermore, bullying rates were lower during the pandemic periods when schools were closed for in-person learning (bullied others during pandemic remote: OR = 0.62, CI = 0.45–0.85; victim of bullying during pandemic remote: OR = 0.24, CI = 0.17–0.34). Children who lived in lower income areas, experienced home life challenges, exhibited mental health difficulties, or had behavioural concerns were more likely to be involved in bullying experiences. Finally, classroom type and school program impacted the child’s likelihood of bullying others or being bullied. These findings further our understanding of the impact of school closures on children’s mental health and behaviour during the pandemic. Public health and policy implications such as bullying prevention, supervision, and conflict management are discussed.

## 1. Introduction

Bullying is the repeated aggression toward another based on power imbalances and leads to physical, social, and/or psychological harm [1]. It is a common problem amongst school-aged children and youth (hereafter referred to as children) and is especially concerning for parents and teachers who hold the responsibility of caring for children [2]. Bullying is a significant issue for students in Ontario, Canada, where 23% of students have reported being bullied during the school year [3]. These rates are similar to the United States where one in five students report being bullied [4]. On a global level, a UNESCO report revealed that 30% of students world-wide have been a victim of bullying [5]. While traditional bullying is face-to-face and can include verbal, physical, or relational tactics [6], children are now faced with a new form of bullying, cyberbullying, because of wide-spread internet use [6]. Cyberbullying typically occurs outside of schools and can involve mobile phones, the internet, and/or text messages [6]. A significant increase in rates of cyberbullying was observed during the COVID-19 pandemic [7,8,9].

Both children who perpetuate bullying, as well as the victims of bullying, are likely to experience negative outcomes. Those who bully their peers in childhood are more likely to engage in a variety of problematic behaviours including aggression, substance abuse, antisocial behaviour, and criminal activity [10,11]. Furthermore, victims of childhood bullying are likely to experience mental health challenges such as depression, anxiety, suicidal ideation, substance abuse, low self-esteem, academic concerns, and difficulties with peer relationships [12,13].

There is also a subset of children who both bully other children and are victims of bullying behaviours. These children are labeled “bully–victims” and are likely to experience social–emotional problems, including internalizing and externalizing symptoms [11,14]. In addition to experiencing greater interpersonal trauma (e.g., physical abuse, neglect, sexual abuse), bully–victims are at increased risk of aggression, anxiety, depression, self-harm, suicidal ideation, and suicide attempts [15].

### 1.1. COVID-19, School Closures, and Bullying

In March of 2020, the COVID-19 pandemic was declared by the World Health Organization (WHO) [16] resulting in school closures, restrictions, and physical distancing precautions to slow the spread of the virus. The closure of schools during the pandemic significantly impacted the social, academic, and psychological outcomes and wellbeing of children [17], especially given that the school environment provides children with important socialization opportunities imperative to their development [17].

Traditional bullying trends during the pandemic showed varied patterns and inconsistent results [18,19]. For example, Bäker and Schütz-Wilke [7] found no significant differences in traditional bullying and externalizing behaviours during the pandemic compared to prior to the pandemic. Conversely, Forsberg and Thorvaldsen [20] found a significant increase in the prevalence of both traditional bullying, conduct problems, and difficulties with peers during the pandemic (vs. pre-pandemic). Similarly, a study in China found the prevalence of bullying perpetration and victimization to increase during the COVID-19 pandemic [21].

Other studies have found that school bullying incidents were lower during the pandemic compared to pre-pandemic levels [22,23,24]. Results from a meta-analysis conducted by Kennedy and Dendy [24] revealed rates of bullying victimization across the United States to be significantly lower during the COVID-19 pandemic from 2020 to 2022 compared to years prior to the pandemic. Comparably, other research conducted with children seeking mental health services found declines in aggression, harm toward others, and bullying during the pandemic, compared to pre-pandemic levels [25]. It is possible that smaller class sizes and increased supervision, coupled with less opportunity for social interaction at school during the pandemic, could explain the decreased bullying rates [22].

### 1.2. Protective and Risk Factors for Bullying

A variety of risk and protective factors, including both individual and relational characteristics, are associated with childhood bullying [22,26]. Studies have found bullying others or experiencing bullying victimization peaks during puberty [20,27] and then decreases thereafter [28]. The child’s sex can also play a role wherein bullying behaviour in boys is more commonly physical and direct, whereas in girls it is more likely relational, online, and indirect [29,30]. Younger male students are also more likely to report bullying others, compared to older female counterparts [22]. Notably, children from low-income, non-traditional families are at heightened risk for being involved in bullying as victims [28,31,32,33].

Risk factors associated with perpetuating bullying include alcohol consumption, smoking, and engaging in videogaming that includes violent content [34,35]. Additionally, sexual and gender minority youth as well as racialized youth experienced higher rates of bullying victimization during the pandemic [22,36]. This may be due to limited resources in certain academic contexts as well as the capacity of personnel to manage the social environment where there are vulnerable students during the pandemic.

During the pandemic, “social” or “physical distancing” was one of the protective measures put in place to help slow the spread of the COVID-19 virus [37]. Researchers have identified that social distancing was a protective barrier against school bullying behaviours. In one study, 78% of students who had experienced bullying before the pandemic were not victimized during the pandemic when social distancing measures were in place [38]. Furthermore, 87% of these students felt safer being in their homes.

A child’s family and home life can play a role in their bullying involvement. As aligned with Bandura’s [39] social learning theory, children learn behaviours from observing the behaviour of others and therefore children exposed to family violence and trauma in childhood are likely to learn negative relationship patterns and see violence to be an acceptable problem-solving technique [26,40]. The literature suggests victims and witnesses of family violence are likely to be victimized by peers and bullied [32,41,42]. Conversely, children who have supportive parents, experience positive parenting practices [12,33], have emotion regulation skills [43], live with both parents [28], and have strong peer friendships [44] are likely more protected from being involved in bullying perpetration or victimization.

Given the literature extant, understanding how school closures played a role in children’s experiences of bullying is needed due to the unprecedented containment measures that reduced access to education, peer contact, and service use. Further research is warranted to understand bullying patterns based on specific roles (e.g., bully, victim, bully–victim) during the COVID-19 pandemic, especially taking into consideration school closures and the timing of data collection [19].

### 1.3. Current Study

The current study utilized a sample of treatment-seeking children who were referred to a mental health agency within Ontario, Canada, and referenced throughout this study as “clinically referred”. Children with mental health concerns and those accessing mental health services are especially vulnerable to the effects of bullying incidents since these children are at an elevated risk of academic difficulties, socio-emotional impairment, substance abuse, and suicidal ideation [45]. Most of the current literature examining COVID-19 within the context of mental health does not compare various timepoints, or compare differences across the pandemic waves, considering school closures. The current study aims to fill this gap by examining bullying while accounting for school closures during the pandemic period within Ontario, Canada. The management and delivery of health care services in the province of Ontario differs from the rest of Canada; therefore, it is important to understand bullying patterns only in Ontario, Canada, which makes up 39% of the Canadian population [46].

It is crucial that researchers continue investigating bullying behaviours during the COVID-19 pandemic as there are significant impacts to children’s wellbeing including their educational, social, and health outcomes [47]. Children included in this study bullied their peers and/or were a victim of bullying. Bullying was defined as a pattern of repeated aggression or victimization of others. It was hypothesized that bullying and victimization would decrease during the COVID-19 pandemic compared to pre-pandemic, and that it would not return to pre-COVID-19 levels until safety measures (e.g., return to school, distancing restrictions) were lifted. It was also hypothesized that behavioural problems would also be lower during the COVID-19 pandemic given that there were added restrictions resulting in the reduction in bullying and victimization.

## 2. Materials and Methods

### 2.1. Sample

Data came from assessments of children and youth receiving mental health services in Ontario, Canada. Assessments were completed as part of regular clinical practice from 43 participating agencies, from January 2017 to February 2022. The assessment instruments are described below. Assessed individuals were referred to these agencies through a variety of sources including family and specialty physicians, school personnel, other allied health professionals, or parents/primary caregivers. Assessment information is used for several purposes, including standardized care planning and the use of items and calculated outcome measures to inform decision making and to track individual change.

The sample included assessed individuals of school age, between the ages of 5 and 18 who were currently enrolled in school, either full or part-time. There were 22,012 individuals in the analytic dataset, using the first assessment if an individual had more than one. Since only the first assessment was used even if the child had more than one, there was no repeat of children’s assessments in the data. The mean age was 12.6 years (SD 3.4) and 51.0% were male.

Assessors completed training in the use of the standardized assessments, and included psychologists, nurses, psychiatrists, speech and language therapists, child and youth workers, developmental social service workers, and social workers. All available sources of information were utilized to complete the assessment (i.e., family members, community members, document review, and clinical observations).

Secure web-based software was implemented to record assessment information, requiring responses of the proper form for all essential items before the record can be authorized as complete. Before making the data available for analysis, personal identifiers were removed. The ethics board granted approval for the secondary analysis of data collected in various agencies throughout the Province of Ontario.

### 2.2. Measures

All measures came from the interRAI Child and Youth Mental Health (ChYMH) or interRAI Child and Youth Mental Health—Developmental Disabilities (ChYMH-DD) assessment items and computed scales. The interRAI ChYMH and ChYMH-DD are semi-structured, interview-based, 400-item assessment instruments developed for use in children and youth 4–18-years-old [48]. The assessment instruments comprehensively assess the physical and mental health of the child, more specifically their strengths, level of functioning, and areas of risk. Various information sources are used by clinicians to complete the assessment including interviews with family members and the child/youth, information from service providers and educators, clinical observation, and previous documentation [48]. Prior to completing the assessments, clinicians receive extensive training on how to administer the interRAI ChYMH and ChYMH-DD. Item-by-item guides are provided to clinicians to assist with interpretation and ensure accurate and standardized assessment across services [48,49]. The results of the assessments are used for research, treatment planning, program evaluation, and resource allocation purposes. Within the assessment instruments are embedded scales and algorithms that have strong construct, concurrent, and predictive validity as well as internal consistency and inter-rater reliability [49,50,51,52,53,54].

#### 2.2.1. Dependent Measures

Bullying peers was defined as a pattern of repeated oppression and victimization of others. It was informed using a single item of the assessment and was coded according to the most recent event; this item was subsequently dichotomized as bullying peers in the last 30 days. Victim of bullying came from another single item of the assessment that records the most recent experience of being bullied by peers. This item was similarly dichotomized as having experienced bullying in the last 30 days.

#### 2.2.2. Independent Measures

The independent measures included school closures, educational classroom setting, and an income variable as described in further detail below.

A 5-level class indicator reflecting how schools were operating on the date of the assessment was assigned according to this hierarchy: (a) school year, pre-pandemic, January 2017 to June 2017, September 2018 June 2019 and September 2019 to June 2020; (b) summer, pre-pandemic, Jul and Aug 2017, 2018, 2019; (c) summer, pandemic, Jul and Aug 2020, 2021; (d) schools operating with remote learning only: 17 March 2020 to 30 June 2020, 8 January 2021 to 16 February 2021, 12 April 2021 to 30 June 2021, and (e) schools open for in-person attendance during the pandemic (remaining times) [55]. We recognized that the bullying measures looked back 30 days from the assessment date, a period that could overlap two different types of school operation. We chose to shift the assessment date back in time 15 days for the assignment of this 5-level indicator which effectively made the 30-day observation window reflect the type of operating period for that was true for the majority of the 30 days. For example, an assessment conducted on 10 July 2020 was treated as if it were conducted on 25 June 2020 (type d) with 20 of the 30 days of look-back time occurring while schools were operating with remote learning only.

Education setting was assigned one of 4 levels: (a) regular classroom without extra support, (b) regular classroom with supports, (c) special school, class, or program, and (d) home-schooled.

In addition, we used the first three digits of the child/youth’s postal code (Forward Sortation Area, FSA) to link to public files of the 2016 Canadian census to inform neighborhood median household income for each FSA [ref: Statistics Canada. Census Profile—Age, sex, type of dwelling, families, households, marital status, language, income, immigration and ethnocultural diversity, housing, aboriginal peoples, education, labour, journey to work, mobility and migration, and language of work for Canada and Forward Sortation Areas, 2016 Census. Ottawa ON, Canada: Government of Canada; 2017. Reference No.: 98-401-X2016046. https://www150.statcan.gc.ca/n1/en/catalogue/98-401-X2016046 (accessed on 1 April 2024). Each FSA represents one of the 513 geographic areas in Ontario, designed for the administration of the postal system. The national statistics agency compiles this information based on the total net income, after taxes, of related individuals residing in the same dwelling, and then calculates the median value within each FSA. We assigned assessments into 3 levels: those below the 25th percentile, those between the 25th and 75th percentiles, and those higher than the 75th percentile.

#### 2.2.3. Scales

Parenting strengths: This scale uses 6 items reflecting parenting activities (communicates effectively, assists with regulation of emotions, appropriate disciplinary practices, demonstrates warmth and support, appropriate supervision, and appropriate limit setting). The items are scored on a scale of 0–2 (where 0 is most of the time, 1 = occasionally, and 2 = rarely or never) and total scores range from 0 to 12, with higher values representing more parenting difficulty [56,57]. For analysis, a cut-point of 3 or more was chosen.

Disruptive/Aggressive Behaviour: As a measure of externalizing behaviours, this sums the presence of 5 behaviours: impulsivity, physically abusive behaviour, outburst of anger, defiance, and argumentativeness [58]. For analysis, all five items needed to be endorsed for reactive aggression to be present. Research found the disruptive and aggressive behaviour scale showed internal consistency, construct validity, and validity in groups of clinically referred children and youth [58].

Internalizing symptoms: The internalizing scale uses 12 items to measure the frequency and severity of internalizing symptoms (anxious complaints, hypervigilance, unrealistic fears, episodes of panic, lack of motivation, anhedonia, withdrawal from activities of interest, decreased energy, negative statements, self-deprecation, expressions of guilt/shame, and expressions of hopelessness), with each item contributing up to 4 points according to frequency in the prior 3 days, for a maximum score of 48 with higher values reflecting more symptoms. For analysis, a cut-point of 11 or more was used. Research shows the internalizing scale to have a strong three-factor structure and good content and concurrent validity [59].

Hyperactive/Distraction: Uses 4 items (impulsivity, distractibility, hyperactivity, disorganization) with each item contributing up to 4 points according to frequency in the prior 3 days, for a maximum score of 16 with higher scores reflecting higher symptoms. For analysis, a cut-point of 6 or more was used. The hyperactive and distraction scale has good internal consistency, construct validity, and validity in groups of clinically referred children and youth [58].

Relational Strengths Index: This Index uses 6 items (reports having a confidant, school engagement, supportive relationship with family, supportive relationship with friends/peers, has at least one friend with whom visits/plays regularly, and social inclusion by peers) with a “no” for each item contributing one point. For the analysis, this was re-coded as 0, 1 and 2, 3 to 6 and a cut point of 3 or more was chosen to represent challenges with relational strengths.

A composite item reflecting the attitude toward the school was created as a binary indicator coded as 1 wherein the child/youth expressed a wish to quit school, or wherein either the child/youth or parent/caregivers felt a strong and persistent dissatisfaction with school.

### 2.3. Analysis

Logistic regression was applied separately to the two dependent variables, bullying peers and victim of bullying, with odds ratios and 95% confidence intervals reported (Supplementary Materials Tables S1 and S2 for details). Univariable and multivariable results were summarized, with the same selected variables used for bullying peers and victim of bullying measures. Additional pairwise models were conducted to test significance between levels of school operation and educational setting classifications. We used *p*-values of 0.05 as denoting statistical significance. SAS version 9.4 was used for all analysis.

## 3. Results

There were 1873 (8.5%) assessments recording victims of bullying in the last month, and 1520 (6.9%) recording bullying of peers. There were 369 (1.7%) that recorded both and 86.3% that recorded neither.

Table 1 summarizes unadjusted rates of bullying, by selected measures. Notably high rates of victims of bullying were found for those with excessive naivete, intention to quit school, or strong persistent dissatisfaction with school, and where parenting difficulties were the greatest. For bullying peers, high rates were found for those denying or minimizing harm to others, with excessive naivete, with high levels of reactive aggression, and with limited understanding of behavioural consequences. Those aged 9 to 11 were most likely to be victims of bullying, and those younger than 11 were about three times as likely to have bullied peers compared to assessed individuals 15 and older. Of note is that bullying rates were lower among those assessed while schools were not open to in-class learning, compared to pandemic periods when schools were open to students.

**Figure 1:**
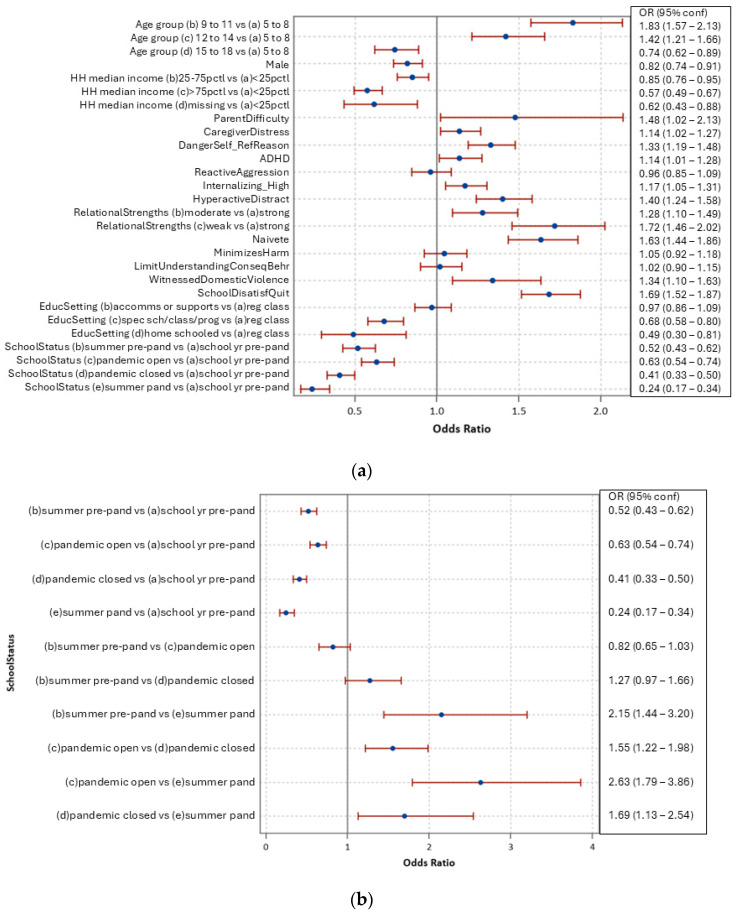

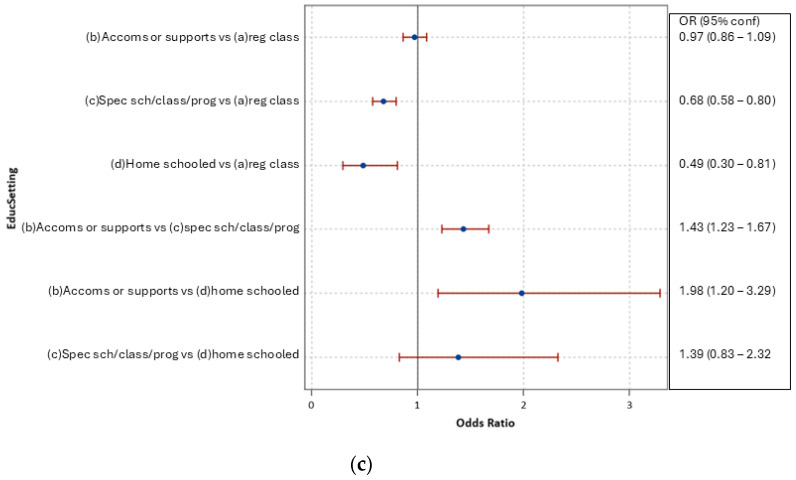
(**a**) Adjusted odds ratios, victim of bullying in the last month. (**b**,**c**) Victim of bullying in last month, adjusted pairwise odds ratios for school/pandemic status and educational setting. Blue dot: point estimate of odds ratio; Red line: 95% confidence interval of odds ratio.

Figure 1a presents adjusted odds ratios of the multivariable model of victim of bullying, using the characteristics listed in Table 1. Notable findings, after adjustment, include higher likelihood of being a victim of bullying among 9- to 14-year-olds, those with parental difficulties or caregiver distress, referred as a risk or danger to self, attention deficit hyperactivity disorder (ADHD) diagnosis, high internalizing behaviours, high levels of hyperactivity/distractibility, fewer relational strengths, having witnessed domestic violence in the last year, or where there is persistent dissatisfaction with school or a desire to quit. Lower adjusted likelihoods were found for those aged 15 or older, male sex, and living in a higher-income neighbourhood.

Figure 1b provides additional adjusted odds ratios of victims of bullying (using the same covariates as Figure 1) for pairwise levels of the school/pandemic status. Of note is that cases assessed during the pandemic when schools were open to in-class learning compared to pre-pandemic periods were less likely to have been a victim of bullying (aOR 0.63, CI 0.54–0.74), and more likely for those during the pandemic when schools were open to in-class learning compared to not open to in-class learning (aOR 1.55, CI 1.22–1.98).

Similarly, Figure 1c provides the pair-wise adjusted odds ratios for type of educational classroom or setting for being a victim of bullying. Those in special schools, classes, or programs as well as those home-schooled were less like to have experienced bullying than those in regular classrooms, with or without accommodations/supports.

**Figure 2:**
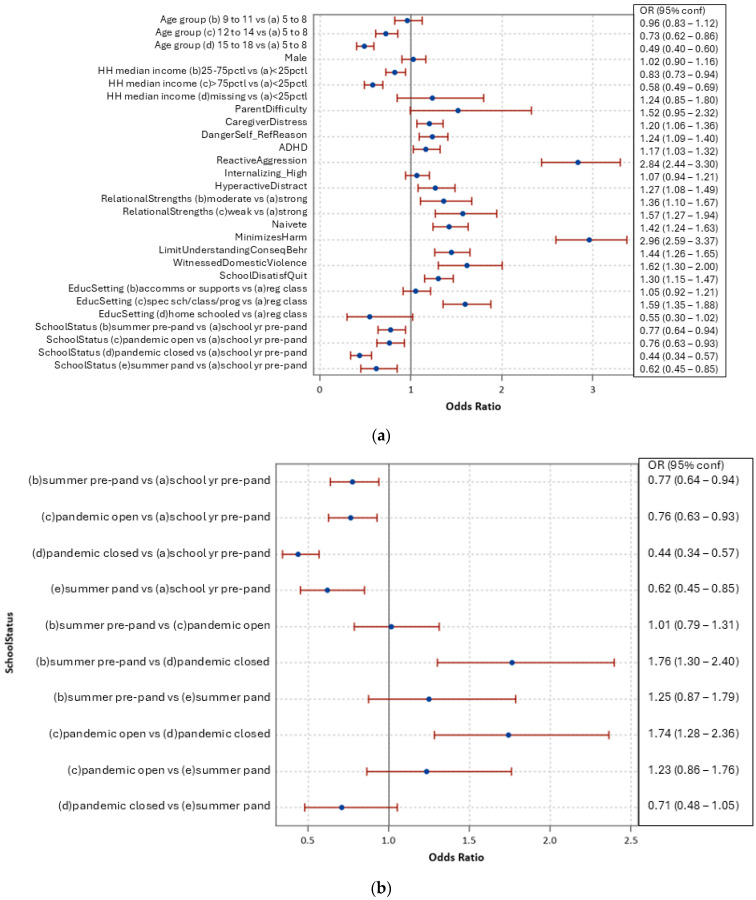

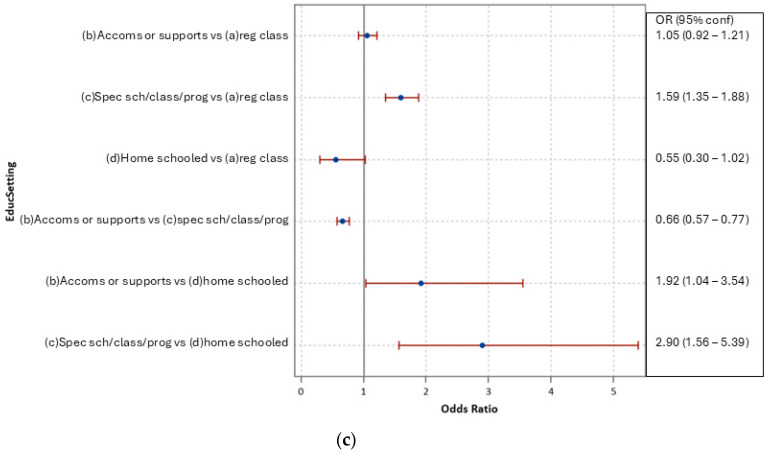
(**a**) Adjusted odds ratios, bullied peers in the last month. (**b**,**c**) Bullied peers in the last month, adjusted pairwise odds ratios for school/pandemic status and educational setting. Blue dot: point estimate of odds ratio; Red line: 95% confidence interval of odds ratio.

Regarding those who bullied peers in the month prior to assessment, Figure 2a shows adjusted odds ratios of the characteristics from Table 1. Higher adjusted likelihood was found for those with greatest parental difficulties, caregiver distress, referred as a threat/risk to self, ADHD, hyperactivity/distractibility, weaker relational strengths, naivete, limited understanding of consequences of their behaviour, having witnessed domestic violence in the last year, or with a strong dissatisfaction with school or intention to quit. Particularly high adjusted likelihoods were observed for those with higher reactive aggressive behaviours and who denied or minimized harm done to others. Lower adjusted likelihood was found for those age 12 or older as well as those living in higher-income neighbourhoods.

Figure 2b reports all pair-wise levels of the school/pandemic status for bullying peers. Here, we observed lower likelihood during pandemic periods when schools were open for in-person learning compared to pre-pandemic periods (aOR 0.76, CI 0.63–0.93) as well as higher during pandemic times when schools were open for in-person learning compared to when they were not open to in-person learning (aOR 1.74, CI 1.28–2.36).

Figure 2c reports pairwise ORs for educational classroom or setting for bullying peers. Those attending special schools, classes, or programs were more likely, after adjustment, to have bullied their peers than in the other three types of settings: regular classrooms with or without supports, or home-schooled.

## 4. Discussion

This study investigated bullying experiences in a sample of clinically referred children (treatment-seeking children who were referred to a mental health agency within Ontario, Canada), as well as how the COVID-19 pandemic and resulting school closures further impacted perpetration and victimization related to bullying. In line with our hypotheses, bullying rates as a bully or victim were lower during the COVID-19 pandemic compared to the pre-pandemic period as well as lower during the pandemic when schools were closed for in-person learning (vs. during the pandemic when schools were open for in-person learning). The type of school classroom or program played a role in children’s bullying experiences. Finally, certain demographic characteristics were associated with increased likelihood of being a bully or experiencing bullying.

### 4.1. Bullying Trends Pre and During the COVID-19 Pandemic

We compared bullying behaviours pre-COVID-19 to during COVID-19 pandemic at the times when schools were open for in-class learning and, as expected, children were less likely to be a victim of bullying or exhibit bullying behaviours during the COVID-19 pandemic period of in-class learning (vs. pre-pandemic). These findings aligned with the current literature showing less aggression [25] and fewer bullying behaviours during the pandemic [22,23]. Consistent with our findings, Vaillancourt and colleagues [22] found higher rates of bullying involvement before the COVID-19 pandemic compared to during the pandemic. Similarly, de Souza and Levandoski [38] found over half of students in their sample who experienced bullying pre-pandemic were not bullied during the pandemic when social distancing measures were in place. It is possible that COVID-19 safety precautions wherein schools had smaller class sizes and were more supervised influenced the lower rates of bullying behaviours during the pandemic. Furthermore, social distancing measures and less social interaction because of distancing measures could have also contributed to these lower rates [22,38].

The findings were consistent with our hypotheses that, during the pandemic when distancing restrictions lifted, and schools were open to in-class learning, compared to virtual learning, children were more likely to be a victim of bullying or exhibit bullying behaviour. Researchers such as de Souza and Levandoski [38] did in fact identify social distancing as a protective factor for experiencing bullying victimization and several students reported feeling safer being in their home. As such, frequent and consistent contact with peers increases the risk of perpetuating bullying or being victimized by bullying behaviours.

It is important to note that our findings contrast some studies showing an increase in the prevalence of bullying during the pandemic compared to before the pandemic [20]. Forsberg and Thorvaldsen [20] conducted a study in Norway investigating child reports of bullying pre-pandemic (2017) and during the pandemic (April 2021) and found an increase in bullying reports during the pandemic. It is possible that varying restrictions and school closures in different countries impacted opportunities for peer interaction and thus bullying rates. Schools and preschools in Norway remained open for most of the pandemic with more flexible measures in place [60]. Researchers highlight this contrast with other countries that closed schools for longer periods of time or had more restrictive measures in place. It is likely that differences in bullying trends during the COVID-19 pandemic are related to differing closures between countries.

Although the closure of in-person learning may have had academic and social disadvantages for children during the pandemic, lower levels of children’s involvement in bullying either as a bully or a victim sheds a positive light on experiences during the pandemic and school closures. It is well known that bullying involvement, both as a victim or a bully, can impact a child’s wellbeing [10,11,12,13], and trends showing a lower level of bullying provides important insights for bullying prevention and intervention efforts. It is important to first note that the pandemic was somewhat of a natural experience in the reduction in bullying levels (compared to pre-pandemic). The increased supervision, social distancing, and early identification of incidents are some of the potential pandemic-related factors that led to these lower bullying rates [19]. Nonetheless, our findings can help researchers, policy makers, and clinicians to better understand the nuanced impact the COVID-19 pandemic had on child well-being.

### 4.2. Bullying and Class Types

Interestingly, our findings regarding bullying roles and the class types are mixed. For those who were a victim of bullying, children in regular classrooms, compared to those in special schools, classes, or programs, and those home-schooled, were more likely to be a victim of bullying. Frequently, children who are perceived to be different are the target of bullying behaviour [61], such as children with disabilities, mental health concerns, behavioural concerns, or additional learning needs [61]. Since children with learning differences, additional needs, or disabilities are often integrated into regular stream classrooms [62], it is possible that high rates of bullying victimization in regular stream classes are reported by these children.

Conversely, for those who bullied others, children attending special schools, classes, or programs were more likely to bully their peers compared to children in regular classroom environments or those home-schooled. It may be that those children placed in specialized classrooms have a variety of neurodevelopmental issues that are often associated with social skills deficits or difficulties. Perceived differences or uniqueness in a specialized group of students (e.g., a class of students with various disabilities, gifted students who vary in their skills and abilities) may increase the risk of bullying behaviours [63]. It could also be a lack of emotional regulation or coping skills in students with additional health needs (e.g., mental health or behavioural concern) that leads to more aggression or bullying behaviours in these groups of children.

The findings regarding bullying involvement and class type highlight the importance of implementing bullying prevention programming broadly to include all schools, and any specialized programs or classes, and not just in regular stream programming, as exhibiting bullying behaviours appear to be more likely in specific types of classrooms. Findings regarding bullying behaviours suggest the importance of implementing bullying prevention programming. This can include but is not limited to enhanced resources for teachers as part of anti-bullying initiatives, greater education related to early signs of bullying (despite a child not appearing distressed), enhanced supervision, contexts to support collaboration amongst students, and structured activities for students to support peer cohesion [10,15,19,64]. It is also possible that children do not display the socio-emotional consequences of bullying at school, but rather hold it in until they return home. Therefore, it is important that parents and other community leaders are involved and aware of the signs of bullying [64] to be able to respond accordingly.

### 4.3. Demographic Factors Associated with Bullying

Several demographic variables were investigated yielding similar results to the previous literature [65]. Children living in higher-income households were less likely to be involved in bullying, compared to lower-income households. Furthermore, children who experienced parental and home life challenges, as well as children with mental health and behavioural concerns, were more likely to be involved in bullying activities as a bully or a victim of bullying. Previous studies have found lower socioeconomic status to be linked to increased rates of bullying [43,65,66]. Children living in lower-income neighbourhoods might lack access to mental health services or community resources that their peers living in higher-income areas may have. It is possible that adolescents in lower-income families may face more adverse living environments because of financial stress, and as a result have more problems with their peers [67]. These adverse living environments may include higher aggression or violence within their families or neighbourhoods, and as a result, these children use learned strategies from their households and communities at school with their peers [40,68]. It is imperative that communities work to increase resources and service access for children in lower-income neighbourhoods. Furthermore, bullying prevention and intervention resources should be prioritized for children in lower-income neighbourhoods.

Similar to previous work [65], both the perpetrators and victims of bullying were found to experience mental health and behavioural concerns, yet victims showed increased internalizing behaviours and bullies had increased levels of reactive aggression, limited understanding of behavioural consequences, and denied or minimized their harmful behaviour toward others. Childhood bullying victimization is associated with depression, anxiety, mood disorders, and suicidal ideation [12,13]. Furthermore, research suggests bullies have more externalizing problems than victims or uninvolved children [11], while also experiencing a high risk of mental health problems, aggression, antisocial behaviours, and harassment [10,11]. These findings emphasize the importance of utilizing trauma-informed approaches in settings working with children involved in bullying, both as a bully or a victim, as it is likely that children involved in bullying incidents have experienced difficult or negative environments and have or may experience mental health concerns. As such, trauma-informed approaches to their clinical care would therefore be essential [69].

### 4.4. Limitations and Future Research Directions

While there are many strengths to this study (e.g., large dataset, comprehensive assessment), one of the limitations of the current study is the sample only consisting of children accessing mental health agencies in Ontario, Canada, and therefore may not be generalizable to non-clinical samples or those living outside of this region. Since differing bullying trends are noted across countries, future research investigating cross-cultural comparisons of bullying trends on an international level during the pandemic is warranted. Furthermore, our study design compared different individuals pre and during COVID-19 pandemic timelines to understand changes in bullying patterns and we did not compare the same individuals at different time points. Future researchers may investigate bullying behaviours in clinical samples comparing the same individual before and during the pandemic to understand within-participant differences. Another limitation of the current study is that online or cyberbullying was not included as a variable. Future research may wish to include this variable to better understand trends in clinical populations across pre- and during-pandemic periods. Additionally, our research did not investigate how children in lower-income neighbourhoods may have been differentially impacted throughout the pandemic. It would be important to understand how global pandemics and crises may impact those who are economically disadvantaged to better prepare and understand how to support these individuals in future crises.

## 5. Conclusions

The current study investigated the influence of the COVID-19 pandemic on bullying experiences in a large clinically referred sample of 22,012 children 4–18-years-old. Related individual, mental health, relational, and community variables were also investigated. Clinically referred children who were more likely to be involved in bullying experiences included individuals living in lower-income households, those experiencing parental and home life challenges, and children with mental health and behavioural concerns. Public health implications for these children include increased bullying screening in schools or community centers, increased supervision, and recreational opportunities for children at risk, and implementing school-based programming [1,70] to teach children emotion regulation skills, empathy, and conflict management. Our finding that bullying rates were lower during the pandemic and when schools were closed to in-person learning suggests the important role of schools in bullying prevention. Standard policies and best practice procedures for responding to bullying incidents, as well as implementing bullying awareness, prevention, and intervention programming, is recommended. Further research should continue to analyze how school closures played a role in children’s bullying behaviours and experiences, especially for children belonging to lower-income households, children with less access to resources and services, and those who are racialized, marginalized, or new to Canada.

## Figures and Tables

**Table 1:** Selected assessment measures and proportions in the last 30 days that were a victim of bullying or bullied peers.

		Last 30 Days	
Measure	N (% of Sample)	Victim of Bullying	Bullied Peers
	22,012	8.5%	6.9%
Age: 5–8	4095 (18.6%)	7.2%	9.8%
9–11	5040 (22.9%)	12.2%	9.8%
12–14	6343 (28.8%)	9.8%	6.4%
15–18	6534 (29.7%)	5.2%	3.3%
Male	11,225 (51.0%)	8.7%	9.0%
Area Household Income: ≤25th percentile	5618 (25.5%)	10.8%	9.5%
between 25th and 75th percentile	11,174 (50.8%)	8.8%	6.8%
>75th percentile	5220 (23.7%)	5.6%	4.2%
Parenting difficulties scale 11+	293 (1.3%)	12.6%	10.6%
Caregiver distress	7172 (32.6%)	11.5%	11.7%
Referred for threat or danger to self	5984 (27.2%)	11.5%	9.8%
ADHD provisional diagnosis	7747 (35.2%)	11.1%	11.9%
Reactive aggression	7319 (33.3%)	11.8%	16.4%
Internalizing behaviours	8701 (39.5%)	10.6%	8.4%
Hyperactivity/Distraction scale 6+	11,696 (53.1%)	10.9%	10.7%
Relational Strengths Scale: 0 (strong)	4476 (20.3%)	5.4%	2.8%
1 or 2 (moderate)	10,745 (48.8%)	7.6%	5.9%
3+ (weak)	6791 (30.9%)	12.0%	11.1%
Demonstrates excessive naivete	2881 (13.1%)	16.4%	17.4%
Denies or minimizes harm done to others	5286 (24.0%)	12.4%	19.7%
Limited understanding of consequences to behaviour	5978 (27.2%)	12.3%	16.2%
Witnessed domestic violence in last year	1064 (4.8%)	12.3%	12.8%
Child/youth or parent strong dissatisfaction with school or child/youth expresses wish to quit school	5967 (27.1%)	13.7%	11.6%
Education status: regular classroom	11,045 (50.2%)	7.4%	4.1%
Reg class with accommodations or supports	7519 (34.2%)	10.4%	8.8%
Special school/class/program	3118 (14.2%)	8.4%	12.9%
Home schooled	330 (1.5%)	5.2%	3.6%
School/pandemic status: pre-pandemic school year	13,142 (59.7%)	10.6%	8.5%
Pre-pandemic, summer months	2180 (9.9%)	6.1%	6.9%
Pandemic, schools open for in-class learning	3123 (14.2%)	6.4%	4.5%
Pandemic, remote learning only	2334 (10.6%)	4.7%	3.1%
Pandemic, summer months	1233 (5.6%)	2.6%	3.8%

## Data Availability

The datasets presented in this article are not readily available due to privacy and confidentiality of the participants and ethical restrictions.

## Supplementary Materials

The following supporting information can be downloaded at: https://www.mdpi.com/article/10.3390/ijerph21121673/s1, Table S1: Victim of bullying in the last month; Table S2: Bullied others in the last month.
