# Supplementary material for: School Closures on Bullying Experiences of Treatment-Seeking Children and Youth: The Influence of the COVID-19 Pandemic Within Ontario, Canada
Source: Int J Environ Res Public Health. 2024 Dec 15;21(12):1673. doi: 10.3390/ijerph21121673 (PMC11675720; doi:10.3390/ijerph21121673)
Supplement: Supplementary file 1 [file ijerph-21-01673-s001.zip › ijerph-3181559-supplementary.pdf]

Supplemental: Multivariable logistic regression

Table S1: Victim of bullying in the last month

	Wald chi-square	p-value	Odds ratio	Lower 95% CL	Higher 95% CL
Age: 5-8 (ref)	167.9	<.0001			
9 – 11			1.83	1.57	2.13
12-14			1.42	1.21	1.66
15-18			0.74	0.62	0.89
Male	13.5	0.0002	0.82	0.74	0.91
Area household income: low (below 25th pctl, ref)	55.1	<.0001			
Mid (between 25th and 75th pctl)			0.85	0.76	0.95
High (>75th pctl)			0.57	0.49	0.67
Missing			0.62	0.43	0.88
Parenting Difficulty Scale (1 or greater vs 0)	4.3	0.0386	1.48	1.02	2.13
Caregiver Distress	5.5	0.0190	1.14	1.02	1.27
Referral reason: danger to self	25.7	<.0001	1.33	1.19	1.48
ADHD diagnosis	4.8	0.0282	1.14	1.01	1.28
Reactive aggression (impulsivity, verbally abusive, outburst of anger, defiant behaviour, argumentative)	0.4	0.5072	0.96	0.85	1.09
High internalizing (scale 11 or greater)	8.5	0.0036	1.17	1.05	1.31
Hyperactive Distractibility Scale 6 or greater	29.5	<.0001	1.40	1.24	1.58
Relational Strengths Scale low (ref)	50.3	<.0001			
Moderate			1.28	1.10	1.49
Strong			1.72	1.46	2.02
Demonstrates excessive naivete	55.0	<.0001	1.63	1.44	1.86
Denies or minimizes harm done to others	0.5	0.4834	1.05	0.92	1.18
Limited understanding of consequences of behaviour	0.1	0.7780	1.02	0.90	1.15
Witnessed domestic violence in the last year	8.2	0.0042	1.34	1.10	1.63
Strong dissatisfaction with school or intent to quit	92.9	<.0001	1.69	1.52	1.87
Education status: regular classroom (ref)	31.4	<.0001			
regular class with accommodations or supports			0.97	0.86	1.09
special school/class/program			0.68	0.58	0.80
home schooled			0.68	0.58	0.80
School/pandemic: school year pre-pandemic (ref)	176.9	<.0001			
summer months, pre-pandemic			0.52	0.43	0.62
summer months, pandemic			0.63	0.54	0.74
pandemic, in-school			0.41	0.33	0.50
pandemic, remote learning			0.24	0.17	0.34
c-statistic	0.722				

Table S2: Bullied others in the last month

	Wald chi-square	p-value	Odds ratio	Lower 95% CL	Higher 95% CL
Age: 5-8 (ref)	64.6	<.0001			
9 – 11			0.96	0.83	1.12
12-14			0.73	0.62	0.86
15-18			0.49	0.40	0.60
Male	0.1	0.7205	1.02	0.90	1.16
Area household income: low (below 25th pctl, ref)	40.8	<.0001			
Mid (between 25th and 75th pctl)			0.83	0.73	0.94
High (>75th pctl)			0.58	0.49	0.69
Missing			1.24	0.85	1.80
Parenting Difficulty Scale (1 or greater vs 0)	3.7	0.0530	1.52	0.95	2.32
Caregiver Distress	8.8	0.0029	1.20	1.06	1.36
Referral reason: danger to self	11.0	0.0009	1.24	1.09	1.40
ADHD diagnosis	5.4	0.0197	1.17	1.03	1.32
Reactive aggression (impulsivity, verbally abusive, outburst of anger, defiant behaviour, argumentative)	180.2	<.0001	2.84	2.44	3.30
High internalizing (scale 11 or greater)	1.0	0.3087	1.07	0.94	1.21
Hyperactive Distractibility Scale 6 or greater	8.3	0.0040	1.27	1.08	1.49
Relational Strengths Scale low (ref)	18.2	0.0001			
Moderate			1.36	1.10	1.67
Strong			1.57	1.27	1.94
Demonstrates excessive naivete	25.9	<.0001	1.42	1.24	1.63
Denies or minimizes harm done to others	261.6	<.0001	2.96	2.59	3.37
Limited understanding of consequences of behaviour	29.5	<.0001	1.44	1.26	1.65
Witnessed domestic violence in the last year	19.2	<.0001	1.62	1.30	2.00
Strong dissatisfaction with school or intent to quit	18.2	<.0001	1.30	1.15	1.47
Education status: regular classroom (ref)	43.2	<.0001			
regular class with accommodations or supports			1.05	0.92	1.21
special school/class/program			1.59	1.35	1.88
home schooled			0.55	0.30	1.02
School/pandemic: school year pre-pandemic (ref)	53.0	<.0001			
summer months, pre-pandemic			0.77	0.64	0.94
summer months, pandemic			0.76	0.63	0.93
pandemic, in-school			0.44	0.34	0.57
pandemic, remote learning			0.62	0.45	0.85
c-statistic	0.852				
